# Exploring quantitative indices to characterize piano timbre with precision validated using measurement system analysis

**DOI:** 10.3389/fpsyg.2024.1363329

**Published:** 2024-06-07

**Authors:** Yuan Zhuang, Shuo Yang

**Affiliations:** ^1^Department of Arts and Media, Tongji University, Shanghai, China; ^2^Department of Biomedical Engineering, Illinois Institute of Technology, Chicago, IL, United States

**Keywords:** piano timbre, timbre characterization, timbre metric, quantitative index, frequency spectrum, precision validation, gage R&R, measurement system analysis

## Abstract

**Aim:**

Timbre in piano performance plays a critical role in enhancing musical expression. However, timbre control in current piano performance education relies mostly on descriptive characterization, which involves large variations of interpretation. The current study aimed to mitigate the limitations by identifying quantitative indices with adequate precision to characterize piano timbre.

**Methods:**

A total of 24 sounds of G6 were recorded from 3 grand pianos, by 2 performers, and with 4 repetitions. The sounds were processed and analyzed with audio software for the frequencies and volumes of harmonic series in the spectrum curves. Ten quantitative timbre indices were calculated. Precision validation with statistical gage R&R analysis was conducted to gage the repeatability (between repetitions) and reproducibility (between performers) of the indices. The resultant percentage study variation (%SV) of an index must be ≤10% to be considered acceptable for characterizing piano timbre with enough precision.

**Results:**

Out of the 10 indices, 4 indices had acceptable precision in characterizing piano timbre with %SV ≤10%, including the square sum of relative volume (4.40%), the frequency-weighted arithmetic mean of relative volume (4.29%), the sum of relative volume (3.11%), and the frequency-weighted sum of relative volume (2.09%). The novel indices identified in the current research will provide valuable tools to advance the measurement and communication of timbre and advance music performance education.

## 1 Introduction

Timbre in music is like color in painting (hence its other name – tone color) and, together with rhythm, melody, and harmony, constitutes the basic elements of music (Copland, 2011). This manifests the critical role that timbre plays in enhancing musical expression. A skilled composer would always carefully select the musical instruments with the ideal timbre he/she needs in a piece of music, and a skilled musician instinctively or intentionally controls the tone color during performances for optimal musical expression. Timbre control may be even more important to pianists, as in many cases they have to perform on and adapt to given pianos rather than their own to create the tone color needed with their performing techniques. The subtle timbre senses and control skills, however, are among the hardest to develop and are usually instilled from years of professional training.

The difficulty lies in the lack of accurate metrics to help measure and communicate timbre. To make communicating timbre even more difficult, due to a lack of sensory vocabulary for auditory experience, currently, musicians or people, in general, rely on descriptive words such as bright, dark, round, dry, harsh, and rich to associate timbre with other sensory or non-sensory attributes (Saitis and Weinzierl, 2019). It is hard to imagine how people would effectively communicate sound pitch with only descriptive words without sound frequency ever being discovered or tuner being invented. Similarly, current ways of communicating timbre bring great limitations and subjectivity.

Prior studies have provided valuable insights in exploring different ways to characterize musical timbre, either qualitatively or quantitatively, as discussed in the later section. However, the question remains to what extent the methods could reliably characterize timbre with subtle differences within a certain musical instrument, such as a piano. Therefore, the current study aimed to explore and discover quantitative indices to precisely characterize piano timbre. The precision of the timbre indices will be validated with the state-of-the-art measurement system analysis methods that have been used in engineering and pharmaceutical industries to ensure the piano timbre measurement system’s reliability and replicability. Note that piano performing techniques, among other factors, can affect the timbre of the sound produced by a piano (Bernays and Traube, 2013, 2014). However, those factors are out of the scope of this study because, without a timbre measurement system with enough precision, semantic associations of timbre are subject to great variation of interpretation (Reymore et al., 2023), and analysis of piano timbre control factors is like shooting for moving (and uncertain) targets. Once the precise timbre indices are identified, they can be used to characterize piano timbre produced with such various factors as performing techniques.

## 2 Materials and methods

Sounds with subtly different timbre from grand pianos were produced and used as standard sounds to identify the timbre indices of enough precision.

### 2.1 Materials and equipment

The materials and equipment used in this study include: three grand pianos: Kawai RX-3 (KWA), Steinway Model A (STW1), and Steinway Model B (STW2); a stainless-steel weight of 120 grams; small amount of clay for cushioning the weight; an audio recorder (Zoom H4n Pro); a computer (Lenovo Thinkpad^®^ X390); sound processing software (WavePad Master Edition v17.28); statistical analysis software (Minitab^®^ 20.2).

### 2.2 Sound recording

**Figure 1:**
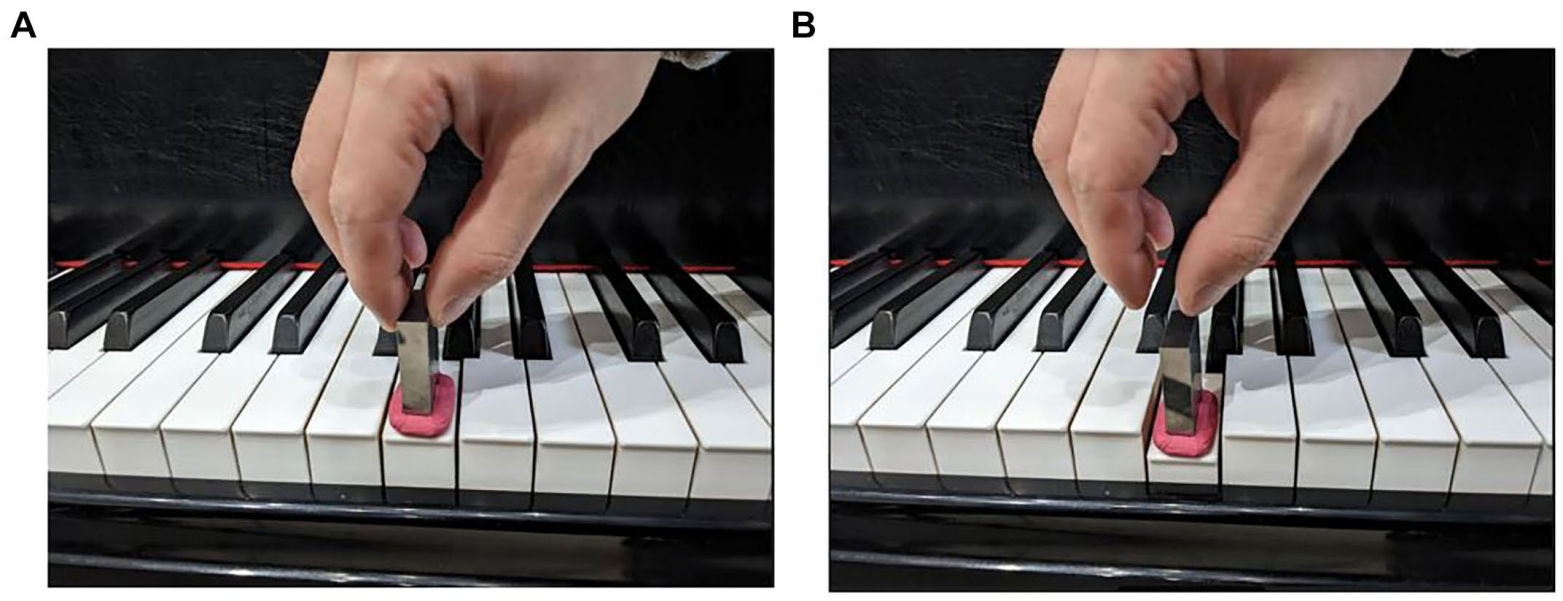
Sound recording by pressing key with a weight. **(A)** The weight was set on the clay, which provided cushioning, and placed on the surface of the key. **(B)** The weight was dropped when the key was pressed to generate the sound.

On each of the three pianos, the G6 key was actuated four times by a performer by releasing the weight at the keyboard level (Figure 1). The performer granted consent for the inclusion of the hand images in Figure 1. The audio recorder was placed near the strings of the key to record the sounds. After the performer finished recording, the second performer recorded the G6 key in the same fashion four times on each of the three pianos. A total of 24 sounds were recorded (4 repetitions × 3 pianos × 2 performers).

### 2.3 Sound processing

**Figure 2:**
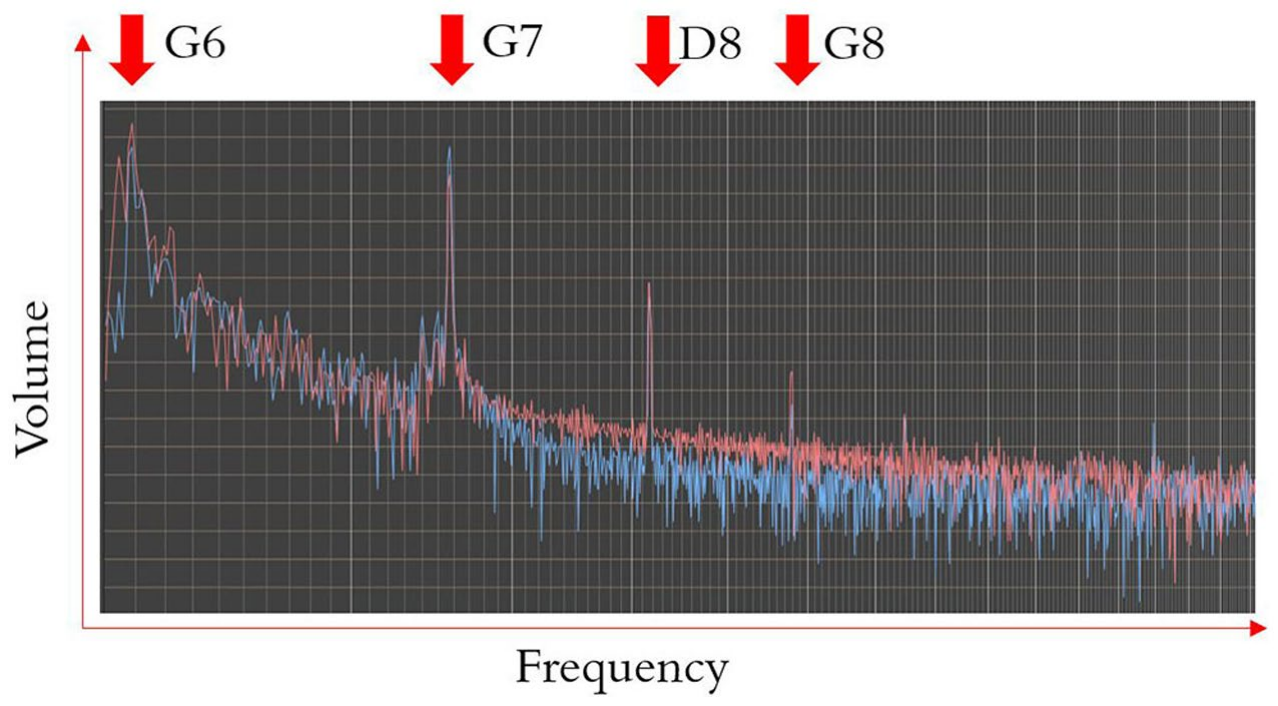
Illustration of the frequency spectrum plot. The red and blue curves are two soundtracks captured from the audio recorder. The peaks of the curves occurred on or near the partials of the harmonic series.

The recorded sounds were imported into WavePad software. The volume peak levels were normalized to −3 dB. The sounds were then transformed from the time domain to the frequency domain with fast Fourier transformation (FFT), see Figure 2. Harmonic series partials were displayed as peaks in the frequency spectrum plot. Volume and frequency data of each partial were collected, with the values from two soundtracks averaged.

### 2.4 Calculation of timbre indices

Ten timbre indices were defined below using the Hz frequency of fundamental (*F_1_*), Hz frequency of partials (*F_n_*), dB volume of fundamental (*V_1_*), dB volume of partials (*V_n_*), and/or number of partials (*N*).

Index 0, Energy integral: $\sum F_{n}\times 10^{0.1V_{n\text{.}}}$

Index 1, Harmonic mean of relative volume: $\frac{N}{\sum \frac{V_{n}}{V_{1}}}$

Index 2, Arithmetic mean of relative volume: $\frac{\sum \frac{V_{1}}{V_{n}}}{N}$

Index 3, Relative volume RMS: $\sqrt{\frac{\sum {\left(\frac{V_{1}}{V_{n}}\right)}^{2}}{N}}$

Index 4, Frequency-weighted arithmetic mean of relative volume: $\frac{\sum \frac{F_{n}V_{1}}{F_{1}V_{n}}}{N}$

Index 5, Frequency-weighted relative volume RMS: $\sqrt{\frac{\sum \frac{F_{n}}{F_{1}}{\left(\frac{V_{1}}{V_{n}}\right)}^{2}}{N}}$

Index 6, Sum of relative volume: $\sum \frac{V_{1}}{V_{n}}$

Index 7, Square sum of relative volume: $\sum {\left(\frac{V_{1}}{V_{n}}\right)}^{2}$

Index 8, Frequency-weighted sum of relative volume: $\sum \frac{F_{n}V_{1}}{F_{1}V_{n}}$

Index 9, Frequency-weighted square sum of relative volume: $\sum \frac{F_{n}}{F_{1}}{\left(\frac{V_{1}}{V_{n}}\right)}^{2}$

### 2.5 Statistical validation of timbre indices

To statistically validate the precision of an index to characterize piano timbre, gage R&R or gage repeatability and reproducibility analysis (Durivage, 2015) was conducted. A good timbre index must consistently measure the timbre of the sounds produced by a performer from the same piano (repeatability) and the timbre of the sounds produced by different performers with the same method from the same piano (reproducibility). The combined variability from repeatability ( $\sigma_{\mathrm{rep}}^{2}$ ) and from reproducibility ( $\sigma_{rpd}^{2}$ ) is the measurement system variability ( $\sigma_{TI}^{2}$ ) of the timbre index, which is expressed as


$$\sigma_{TI}^{2}=\sigma_{\mathrm{rep}}^{2}+\sigma_{rpd}^{2}$$

A good timbre index must also be able to differentiate the timbre of the sounds from piano to piano without drastically interfered by the timbre index measurement system’s uncertainty. In other words, the timbre index measurement system variability ( $\sigma_{TI}^{2}$ ), as noise, must be sufficiently smaller than the piano-to-piano variability ( $\sigma_{\mathrm{piano}-to-\mathrm{piano}}^{2}$ ), as a signal. This can be evaluated with percent study variation (%SV), which is defined as


$$\%SV=\%100\times \frac{\sqrt{\sigma_{TI}^{2}}}{\sqrt{\sigma_{TI}^{2}+\sigma_{\mathrm{piano}-to-\mathrm{piano}}^{2}}}$$

If a timbre index %SV is ≤ 10%, indicating that minimal variation is from repetitions or performers (noise) versus pianos of different timbres (signal), the index has acceptable piano timbre characterization capability. If the timbre index %SV is >30%, the index is unacceptable to characterize piano timbre. If the timbre index %SV is >10% and ≤ 30%, the index has marginally acceptable piano timbre characterization capability.

## 3 Results

### 3.1 Volume and frequency of sounds

**Figure 3:**
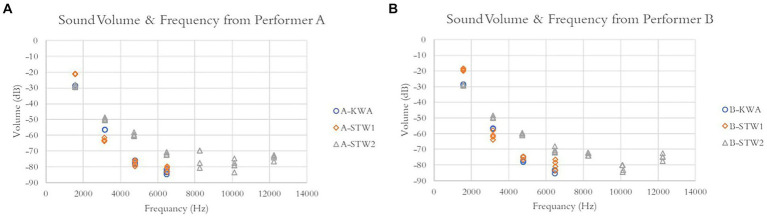
Volume–frequency plots of the recorded sounds. **(A)** Twelve sounds are recorded by performer A. **(B)** Twelve sounds are recorded by performer B.

Volume and frequency data of the harmonic series partials from the 24 recorded sounds are plotted in Figure 3. Sounds from each of the two performers (A and B) were plotted in separate charts. The plots showed that the volume and frequency data from the two performers were similar. Data within partials were consistent.

The volume–frequency plots showed different patterns for the sounds from different pianos. KWA and STW1 piano sounds showed four partials, ranging from 1,600 Hz to 6,500 Hz, and STW2 piano sounds showed seven partials, ranging from 1,600 Hz to 12,200 Hz. The volume of overtone partials relative to the volume of the fundamental partial was also different from piano to piano. These differences determined the timbre of the sound.

### 3.2 Index performances in timbre characterization

Timbre of the 24 recorded sounds from 3 pianos, 2 performers, and 4 repetitions are characterized by the indices defined in Materials and Methods section 2.4. The indices results for all 24 recorded sounds are provided in Supplementary material for this article.

Gage R&R analysis was performed for each index, and the resultant %SV is presented in Table 1. Two indices were identified to have unacceptable timbre characterization precision (%SV > 30%): Index 5 (frequency-weighted relative volume RMS) and Index 9 (frequency-weighted square sum of relative volume). Four indices were identified to have marginally acceptable timbre characterization precision (10% < %SV ≤3 0%): Index 0 (energy integral), Index 1 (harmonic mean of relative volume), Index 2 (arithmetic mean of relative volume), and Index 3 (relative volume RMS). The other four indices were identified to have acceptable timbre characterization precision (%SV ≤ 10%): Index 4 (frequency-weighted arithmetic mean of relative volume), Index 6 (sum of relative volume), Index 7 (square sum of relative volume), and Index 8 (frequency-weighted sum of relative volume).

**Table 1:** Percent study variation of timbre indices.

**Unacceptable**	**Index 5**	**Index 9**		
%SV	93.12	99.43		
**Marginally acceptable**	**Index 0**	**Index 1**	**Index 2**	**Index 3**
%SV	15.00	12.06	12.84	10.76
**Acceptable**	**Index 4**	**Index 6**	**Index 7**	**Index 8**
%SV	4.29	3.11	4.40	2.09

**Figure 4:**
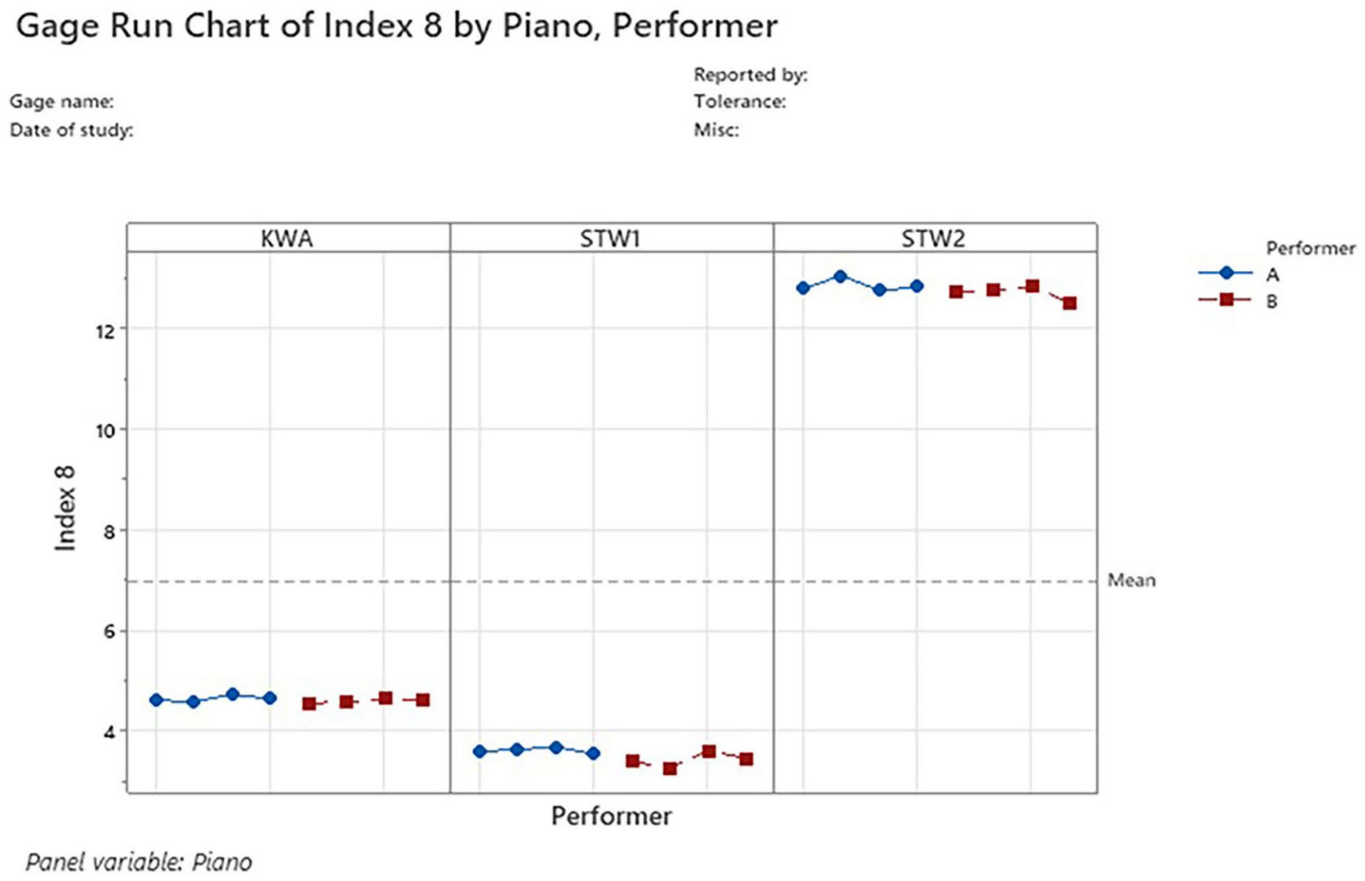
Index 8 gage run chart. The timbre measurements for KWA, STW1, and STW2 pianos are plotted in the left, middle, and right panels, respectively. Measurements from each of the three pianos are consistent, indicating good repeatability. Measurements between the sounds from performers A and B are consistent, indicating good reproducibility.

Among the four indices with acceptable timbre characterization precision, Index 8 (frequency-weighted sum of relative volume) had the best performance, with the lowest %SV, indicating that it differentiated the timbre of the sounds from piano to piano with minimal measurement system variability, as shown in the gage run chart in Figure 4. Index 8 measured KWA piano timbre at 4.609±0.056 , STW1 piano timbre at 3.512±0.141 , and STW2 piano timbre at 12.792±0.149 .

**Figure 5:**
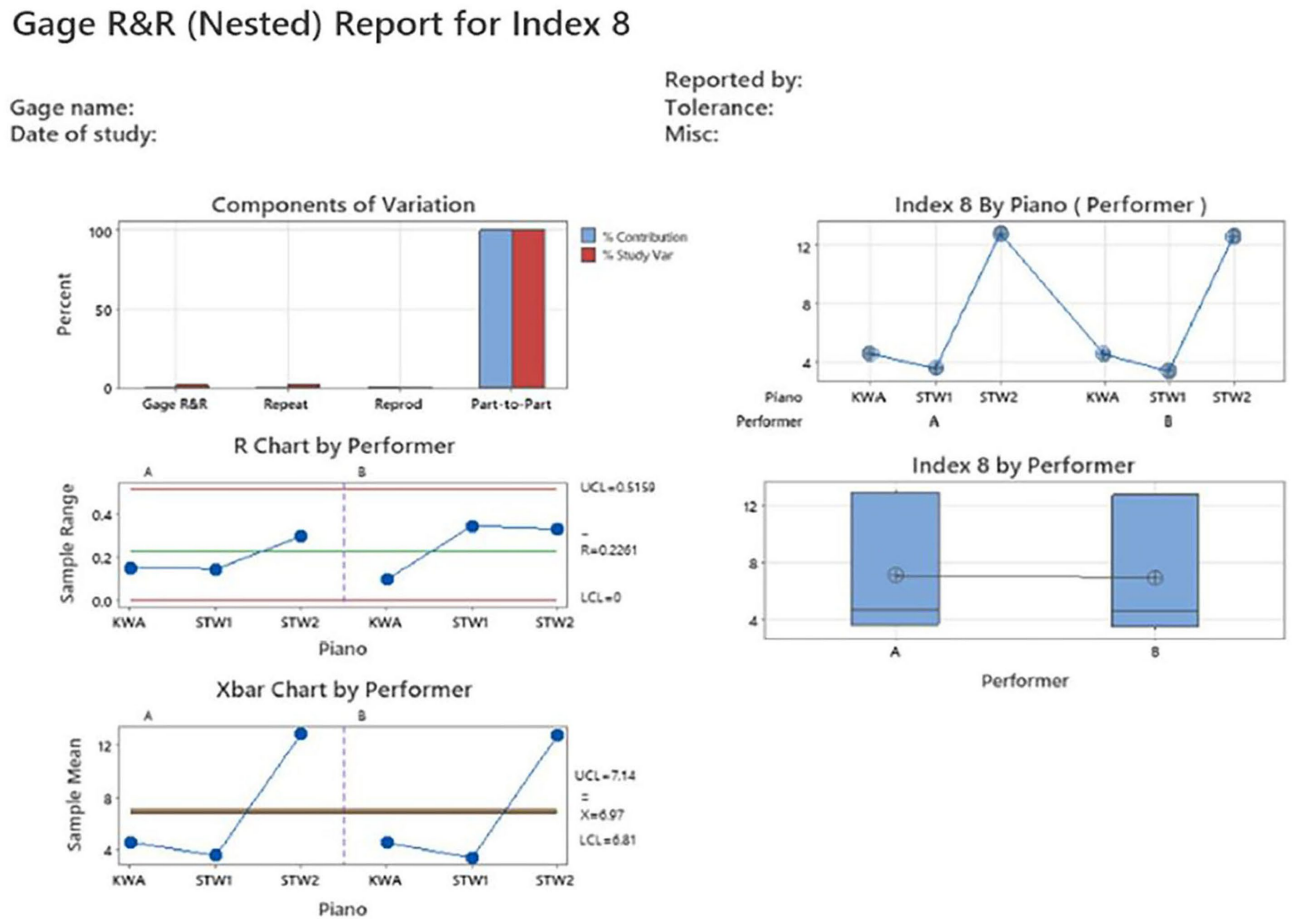
Gage R&R analysis report for Index 8 frequency-weighted sum of relative volume. The %SV result was 2.09 (see components of variation chart), indicating acceptable precision. R chart has all points falling within UCL and LCL, indicating repeatability is good. Xbar chart showed points spreading well beyond the UCL and LCL, indicating that Index 7 can well differentiate timbre from different pianos. Index 7 by performer chart and Index 7 by piano (performer) chart showed almost identical results between performers A and B, indicating reproducibility is good.

Gage R&R study results for Index 8 are presented in Table 2 and Figure 5. The %SV result was 2.09 (see gage evaluation in Table 2). The variability from the Index 8 measurement system accounted for only 0.04% of the total variability, while the piano-to-piano variability accounted for 99.96% (see variance components in Table 2 and components of variation chart in Figure 5). R chart in Figure 5 shows the range of timbre values (maximum – minimum) from the repeated measurements of a piano by a performer. All points in the R chart were within the upper control limit and lower control limit (UCL and LCL), indicating good repeatability (i.e., the repeated measurements from the same piano were well controlled). The Xbar chart in Figure 5 shows the average of timbre values from the repeated measurements of a piano by a performer. The points in the Xbar chart spread well beyond the UCL and LCL, indicating that Index 8 can well differentiate timbre from different pianos. Furthermore, the patterns between performers A and B were similar, as shown in the Xbar chart and Index 8 by piano (performer) chart, indicating good reproducibility (i.e., different people pressing the same piano key in the same way generated similar Index 8 values). Therefore, Index 8 has adequate precision in characterizing piano timbre.

**Table 2:** Gage R&R study results for Index 8—nested ANOVA.

Gage R&R (nested) for Index 8					
Source	DF	SS	MS	F	P
Performer	1	0.094	0.094	0.00	0.977
Piano (Performer)	4	411.437	102.859	9132.97	0.000
Repeatability	18	0.203	0.011		
Total	23	411.733			

Variance components
Source	VarComp	%Contribution (of VarComp)
Total gage R&R	0.0113	0.04
Repeatability	0.0113	0.04
Reproducibility	0.0000	0.00
Part-to-part	25.7120	99.96
Total variation	25.7232	100.00

Gage evaluation					
Source	StdDev (SD)	Study Var (6 × SD)	%Study Var (%SV)
Total gage R&R	0.10612	0.6367	2.09
Repeatability	0.10612	0.6367	2.09
Reproducibility	0.00000	0.0000	0.00
Part-to-part	5.07070	30.4242	99.98
Total variation	5.07181	30.4308	100.00

Number of distinct categories = 67.

Gage R&R analysis results for Index 0–9 except Index 8 are presented in Supplementary material for this article.

**Figure 6:**
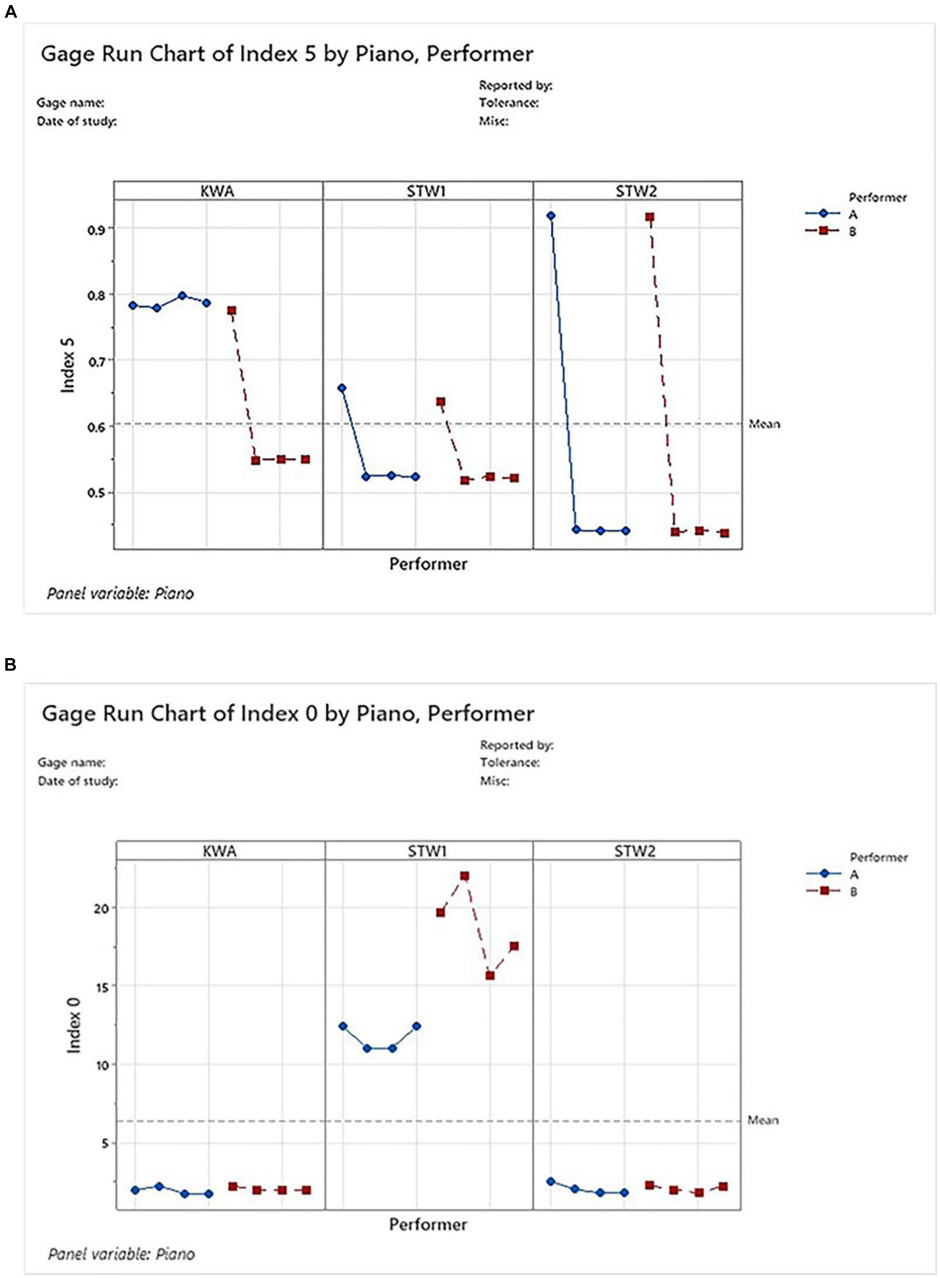
Gage run charts for Index 5 and Index 0. **(A)** Index 5 had timbre characterization results vary within a performer for each of the three pianos and vary between performers within a piano, indicating unacceptable precisions. **(B)** Index 0 had results that varied dramatically between the performers and within performer B in STW1 piano, which undermined the precision of index 0.

By contrast, indices with unacceptable precision (represented by Index 5) and marginally acceptable precision (represented by Index 0, see Figure 6 and Supplementary Figure S1) do not have as good repeatability or reproducibility as the indices with acceptable precision. The gage run charts for Index 5 and Index 0 (Figure 6) showed that timbre characterization results within a performer for a piano and results between the performers within a piano vary dramatically, compared to the consistent results within a piano in the gage run charts for Index 8 (Figure 4). Gage R&R analysis for Index 5 showed that most variations of the results were from the gage rather than from part-to-part (i.e., between the pianos) (see Supplementary Figure S6 components of variation), and the lack of precision in the gage failed to differentiate the timbre differences in different pianos (see Supplementary Figure S6 Xbar chart). Gage R&R analysis for Index 0 showed that although most variations were from the part-to-part instead of the gage, the repeatability within the STW1 piano was out of control, as manifested by the STW1 point went well above the UCL (Supplementary Figure S1 R chart). Those gage R&R results for Index 5 and Index 0 showed significant contrasts when compared to the results for Index 8 with acceptable precision (Figure 5).

In conclusion, the indices with acceptable precisions, as validated by gage R&R, provide reliable ways to characterize the piano timbre.

## 4 Discussions

Modern timbre analysis began in the mid-1970s when spectrograms became widely available to allow researchers visually see the sounds. Cogan’s pioneering studies combined spectrogram analysis with a series of oppositions that can describe sound features of a given sound signal (Cogan, 1984). After that, timbre analysis gradually became a hot research topic, and the analysis methods developed into two large categories: qualitative or semi-quantitative methods and quantitative methods. Many prior studies used qualitative or semi-quantitative methods to characterize timbre and associate it with semantic meanings (e.g., Petiot et al., 2017; Kazazis et al., 2021; Reymore, 2022; Reymore et al., 2023). Other studies proposed various mathematical models developed for identifying the timbre of different musical instruments (McAdams et al., 2017; Thoret et al., 2017; Jiang et al., 2020; Jannereth and Esch, 2021). However, a common challenge is that the precision of the measurement system to characterize timbre has not been statistically validated for repeatability and reproducibility. Therefore, it is not clear to what extent the timbre characterization results following those methods are reliable and replicable. Thoret et al. (2021) computationally re-analyzed 17 datasets from studies published between 1977 and 2016 to correlate timbre features with various instrument sources and observed that original results were only partially replicable. Furthermore, within an instrument, timbre varies more subtly yet meaningfully to musical expression, and quantitative characterization with adequate precision for timbre within a specific instrument, such as a piano, becomes valuable. The current study aimed to take on the challenges by quantitatively exploring multiple mathematical indices for piano timbre and rigorously validating the precision of the indices, with state-of-the-art measurement system analysis method of gage R&R from the engineering and pharmaceutical industries, to ensure the indices’ capability of characterizing timbre within pianos with subtle timbre differences. The novel indices identified and validated to have acceptable precisions in the current research will prove to be valuable tools to advance the measurement and communication of piano timbre.

As the use of the tuner could greatly help novice violinists without the sense of perfect pitch develop accurate pitch playing, the application of the piano timbre indices could greatly advance piano performance education by bridging the performing techniques and timbre outputs. One of the difficulties in piano performance study is to receive timely and accurate feedback during practice, and one cannot always expect students to develop accurate sense of timbre for ideal musical expression. The timbre indices, especially integrated with music education software, could provide valuable feedback to aid ideal timbre control.

One limitation of the current research is that the indices were only validated to have adequate timbre characterization precision for pianos. This will limit the application of the indices. Many musical instruments widely used in solo performance or orchestration, such as violin, cello, clarinet, and flute have different timbre characteristics between instruments and valuable timbre expressions within an instrument. Different timbre between instruments is of great concern to composers to decide which instruments to use in a music piece. This has largely been studied and perfected throughout time. It is the timbre subtleness within an instrument that is of utmost importance for musical expression from performance and merit more research in reliable characterization. The validities of the timbre indices investigated in the current research need to be studied for applicability in those other instruments. Through those studies, potential universal timbre indices to characterize musical timbre may be discovered.

Another limitation of the current research is that the relations between the timbre indices quantities and common timbre descriptions have not been explored. This will limit the understanding of the meaning of the timbre indices, without which effective communication of timbre for ideal musical expression of a note or a music piece will be limited. The issue relates to timbre perception and cognition in human sensory and central nervous organs. It is possible that some subtle differences in timbre in a certain instrument may not be recognizable by the audience but may be detected by the timbre precision measurement system, in which case the detected differences do not carry much musical meaning. There may be thresholds of timbre differences, beyond which trained musicians and the general audience may recognize the musical expression differences and regions of timbre quantities that are associated with semantic meanings. Therefore, research in timbre perception on the scale of detectable differences and relation analysis between timbre expression and indices are merited in future studies. Those studies will greatly advance the understanding and effective communication of the musical timbre.

## Data availability statement

The original contributions presented in the study are included in the article/Supplementary material, further inquiries can be directed to the corresponding author.

## Author contributions

YZ: Conceptualization, Formal analysis, Funding acquisition, Investigation, Methodology, Project administration, Resources, Validation, Writing – original draft, Writing – review & editing. SY: Formal analysis, Investigation, Methodology, Software, Validation, Writing – original draft, Writing – review & editing.

## References

[ref1] Bernays M. Traube C. (2013). Expressive production of piano timbre: touch and playing techniques for timbre control in piano performance. 10th sound and music computing conference (SMC2013), Stockholm, Sweden.

[ref2] Bernays M. Traube C. (2014). Investigating pianists' individuality in the performance of five timbral nuances through patterns of articulation, touch, dynamics, and pedaling. Front. Psychol. 5:157. doi: 10.3389/fpsyg.2014.00157, PMID: 24624099 PMC3941302

[ref3] Cogan R. (1984). New images of musical sound. Cambridge, MA: Harvard University Press.

[ref4] Copland A. (2011). What to listen for in music. New York, NY: Signet Classics.

[ref5] Durivage M. A. (2015). Practical attribute and variable measurement systems analysis (MSA): A guide for Conducting Gage R&R Studies and test method validations. Milwaukee, WI: ASQ Quality Press.

[ref6] Jannereth E. Esch L. (2021). Analyzing timbres of various musical instruments using FFT and spectral analysis. J. Stud. Res. 10, 1–9. doi: 10.47611/jsrhs.v10i1.1292

[ref7] Jiang W. Liu J. Zhang X. Wang S. Jiang Y. (2020). Analysis and modeling of timbre perception features in musical sounds. Appl. Sci. 10:789. doi: 10.3390/app10030789

[ref8] Kazazis S. Depalle P. McAdams S. (2021). Ordinal scaling of timbre-related spectral audio descriptors. J. Acoust. Soc. Am. 149, 3785–3796. doi: 10.1121/10.0005058, PMID: 34241417

[ref9] McAdams S. Douglas C. Vempala N. N. (2017). Perception and modeling of affective qualities of musical instrument sounds across pitch registers. Front. Psychol. 8:153. doi: 10.3389/fpsyg.2017.00153, PMID: 28228741 PMC5296353

[ref10] Petiot J. F. Kersaudy P. Scavone G. McAdams S. Gazengel B. (2017). Investigation of the relationships between perceived qualities and sound parameters of saxophone reeds. Acta Acust. 103, 812–829. doi: 10.3813/AAA.919110

[ref11] Reymore L. (2022). Characterizing prototypical musical instrument timbres with timbre trait profiles. Music. Sci. 26, 648–674. doi: 10.1177/10298649211001523

[ref12] Reymore L. Noble J. Saitis C. Traube C. Wallmark Z. (2023). Timbre semantic associations vary both between and within instruments: an empirical study incorporating register and pitch height. Music. Percept. 40, 253–274. doi: 10.1525/mp.2023.40.3.253

[ref13] Saitis C. Weinzierl S. (2019). “The semantics of timbre” in Timbre: Acoustics, perception, and cognition. eds. Siedenburg K. Saitis C. McAdams S. Popper A. N. Fay R. R. (Cham, Switzerland: Springer), 119–149.

[ref14] Thoret E. Caramiaux B. Depalle P. McAdams S. (2021). Learning metrics on spectrotemporal modulations reveals the perception of musical instrument timbre. Nat. Hum. Behav. 5, 369–377. doi: 10.1038/s41562-020-00987-5, PMID: 33257878

[ref15] Thoret E. Depalle P. McAdams S. (2017). Perceptually salient regions of the modulation power Spectrum for musical instrument identification. Front. Psychol. 8:587. doi: 10.3389/fpsyg.2017.00587, PMID: 28450846 PMC5390014

