# Supplementary material for: Exploring quantitative indices to characterize piano timbre with precision validated using measurement system analysis
Source: Front Psychol. 2024 Jun 7;15:1363329. doi: 10.3389/fpsyg.2024.1363329 (PMC11204798; doi:10.3389/fpsyg.2024.1363329)
Supplement: Supplementary file 1 [file Data_Sheet_1.PDF]

frontiers

Supplementary Material

Exploring Quantitative Indices to Characterize Piano Timbre with Precision Validated Using Measurement System Analysis

Yuan Zhuang*, Shuo Yang

*** Correspondence:** Yuan Zhuang: yuanzhuang@tongji.edu.cn

1 Supplementary Data

The frequency and volume raw data of all 24 recorded sounds are provided below to support reproducing analyses.

**Performer A**	**Repetition 1**		**Repetition 2**		**Repetition 3**		**Repetition 4**	
**Piano: KWA**	**Freq. (Hz)**	**Vol. (dB)**	**Freq. (Hz)**	**Vol. (dB)**	**Freq. (Hz)**	**Vol. (dB)**	**Freq. (Hz)**	**Vol. (dB)**
	1572	-29	1572	-29	1572	-30	1572	-30
	3164	-57	3166	-57	3164	-57	3164	-57
	4791	-78	4791	-76	4783	-76	4791	-79
	6467	-84	6470	-83	6469	-82	6473	-85
**Performer A**	**Repetition 1**		**Repetition 2**		**Repetition 3**		**Repetition 4**	
**Piano: STW1**	**Freq. (Hz)**	**Vol. (dB)**	**Freq. (Hz)**	**Vol. (dB)**	**Freq. (Hz)**	**Vol. (dB)**	**Freq. (Hz)**	**Vol. (dB)**
	1562	-21	1562	-22	1562	-22	1562	-21
	3146	-64	3146	-64	3146	-62	3146	-63
	4771	-79	4771	-76	4771	-77	4771	-80
	6502	-80	6502	-83	6502	-81	6500	-83
**Performer A**	**Repetition 1**		**Repetition 2**		**Repetition 3**		**Repetition 4**	
**Piano: STW2**	**Freq. (Hz)**	**Vol. (dB)**	**Freq. (Hz)**	**Vol. (dB)**	**Freq. (Hz)**	**Vol. (dB)**	**Freq. (Hz)**	**Vol. (dB)**
	1562	-28	1562	-29	1562	-30	1562	-30
	3150	-49	3150	-49	3150	-50	3150	-51
	4716	-60	4716	-58	4716	-60	4716	-61
	6478	-71	6481	-73	6481	-72	6481	-71
	8250	-70	8250	-70	8250	-81	8250	-78
	10108	-75	10108	-78	10108	-84	10112	-79
	12240	-73	12240	-74	12240	-73	12240	-77
**Performer B**	**Repetition 1**		**Repetition 2**		**Repetition 3**		**Repetition 4**	
**Piano: KWA**	**Freq. (Hz)**	**Vol. (dB)**	**Freq. (Hz)**	**Vol. (dB)**	**Freq. (Hz)**	**Vol. (dB)**	**Freq. (Hz)**	**Vol. (dB)**
	1572	-29	1572	-29	1572	-29	1573	-29
	3164	-57	3166	-57	3164	-57	3164	-57
	4782	-77	4782	-77	4783	-75	4782	-78

	6467	-84	6470	-86	6474	-83	6473	-84
**Performer B**	**Repetition 1**		**Repetition 2**		**Repetition 3**		**Repetition 4**	
**Piano: STW1**	**Freq. (Hz)**	**Vol. (dB)**	**Freq. (Hz)**	**Vol. (dB)**	**Freq. (Hz)**	**Vol. (dB)**	**Freq. (Hz)**	**Vol. (dB)**
	1562	-19	1562	-19	1562	-20	1562	-20
	3146	-61	3146	-64	3146	-58	3146	-62
	4769	-75	4771	-75	4771	-75	4771	-77
	6496	-81	6502	-84	6499	-77	6500	-79
**Performer B**	**Repetition 1**		**Repetition 2**		**Repetition 3**		**Repetition 4**	
**Piano: STW2**	**Freq. (Hz)**	**Vol. (dB)**	**Freq. (Hz)**	**Vol. (dB)**	**Freq. (Hz)**	**Vol. (dB)**	**Freq. (Hz)**	**Vol. (dB)**
	1562	-29	1562	-29	1562	-30	1562	-29
	3150	-49	3150	-50	3150	-50	3150	-50
	4716	-60	4716	-60	4716	-61	4716	-60
	6478	-72	6481	-72	6481	-71	6470	-68
	8250	-72	8267	-74	8250	-74	8264	-73
	10078	-80	10108	-80	10108	-85	10112	-83
	12240	-73	12240	-75	12240	-75	12240	-78

2 Supplementary Tables and Figures

2.1 Supplementary Tables

Calculated results of Index 0-9 for all 24 recorded sounds are provided below.

Supplementary Table 1. Index Results for 24 Recorded Sounds

Piano	Performer	Repetition	Index 0	Index 1	Index 2	Index 3	Index 4
KWA	A	1	1.986	0.471	0.559	0.617	1.151
KWA	A	2	2.228	0.467	0.556	0.615	1.143
KWA	A	3	1.771	0.485	0.568	0.623	1.180
KWA	A	4	1.771	0.474	0.562	0.620	1.158
STW1	A	1	12.409	0.346	0.466	0.559	0.896
STW1	A	2	11.060	0.352	0.470	0.562	0.906
STW1	A	3	11.061	0.358	0.474	0.564	0.919
STW1	A	4	12.409	0.341	0.463	0.558	0.883
STW2	A	1	2.528	0.463	0.515	0.556	1.830
STW2	A	2	2.016	0.473	0.525	0.564	1.866
STW2	A	3	1.790	0.460	0.516	0.558	1.823
STW2	A	4	1.786	0.465	0.519	0.559	1.834
KWA	B	1	2.228	0.464	0.554	0.614	1.136
KWA	B	2	1.985	0.467	0.556	0.616	1.142
KWA	B	3	1.986	0.475	0.561	0.619	1.160
KWA	B	4	1.987	0.470	0.558	0.617	1.148
STW1	B	1	19.667	0.323	0.450	0.552	0.845

STW1	B	2	22.065	0.307	0.439	0.546	0.814
STW1	B	3	15.625	0.349	0.469	0.561	0.901
STW1	B	4	17.528	0.330	0.454	0.554	0.861
STW2	B	1	2.257	0.461	0.515	0.557	1.820
STW2	B	2	2.004	0.462	0.516	0.557	1.824
STW2	B	3	1.793	0.465	0.520	0.561	1.835
STW2	B	4	2.245	0.454	0.510	0.553	1.788
**Piano**	**Performer**	**Repetition**	**Index 5**	**Index 6**	**Index 7**	**Index 8**	**Index 9**
KWA	A	1	0.783	2.235	1.524	4.602	2.453
KWA	A	2	0.779	2.223	1.513	4.572	2.426
KWA	A	3	0.798	2.272	1.554	4.721	2.546
KWA	A	4	0.788	2.247	1.536	4.634	2.481
STW1	A	1	0.658	1.862	1.251	3.583	1.729
STW1	A	2	0.524	1.881	1.262	3.624	1.099
STW1	A	3	0.525	1.898	1.273	3.674	1.104
STW1	A	4	0.523	1.850	1.245	3.531	1.093
STW2	A	1	0.920	3.607	2.163	12.807	5.926
STW2	A	2	0.442	3.675	2.228	13.059	1.369
STW2	A	3	0.441	3.615	2.180	12.763	1.359
STW2	A	4	0.440	3.630	2.187	12.836	1.358
KWA	B	1	0.776	2.216	1.508	4.545	2.408
KWA	B	2	0.548	2.225	1.516	4.566	1.203
KWA	B	3	0.549	2.245	1.530	4.639	1.207
KWA	B	4	0.549	2.232	1.522	4.592	1.206
STW1	B	1	0.637	1.801	1.217	3.382	1.623
STW1	B	2	0.518	1.757	1.193	3.258	1.073
STW1	B	3	0.524	1.875	1.259	3.602	1.099
STW1	B	4	0.521	1.818	1.226	3.446	1.085
STW2	B	1	0.918	3.606	2.168	12.742	5.894
STW2	B	2	0.439	3.610	2.169	12.770	1.352
STW2	B	3	0.441	3.639	2.200	12.845	1.364
STW2	B	4	0.438	3.572	2.138	12.516	1.344

2.2 Supplementary Figures

Precision Validation Gage R&R reports for index 0-7 and index 9 are provided below.

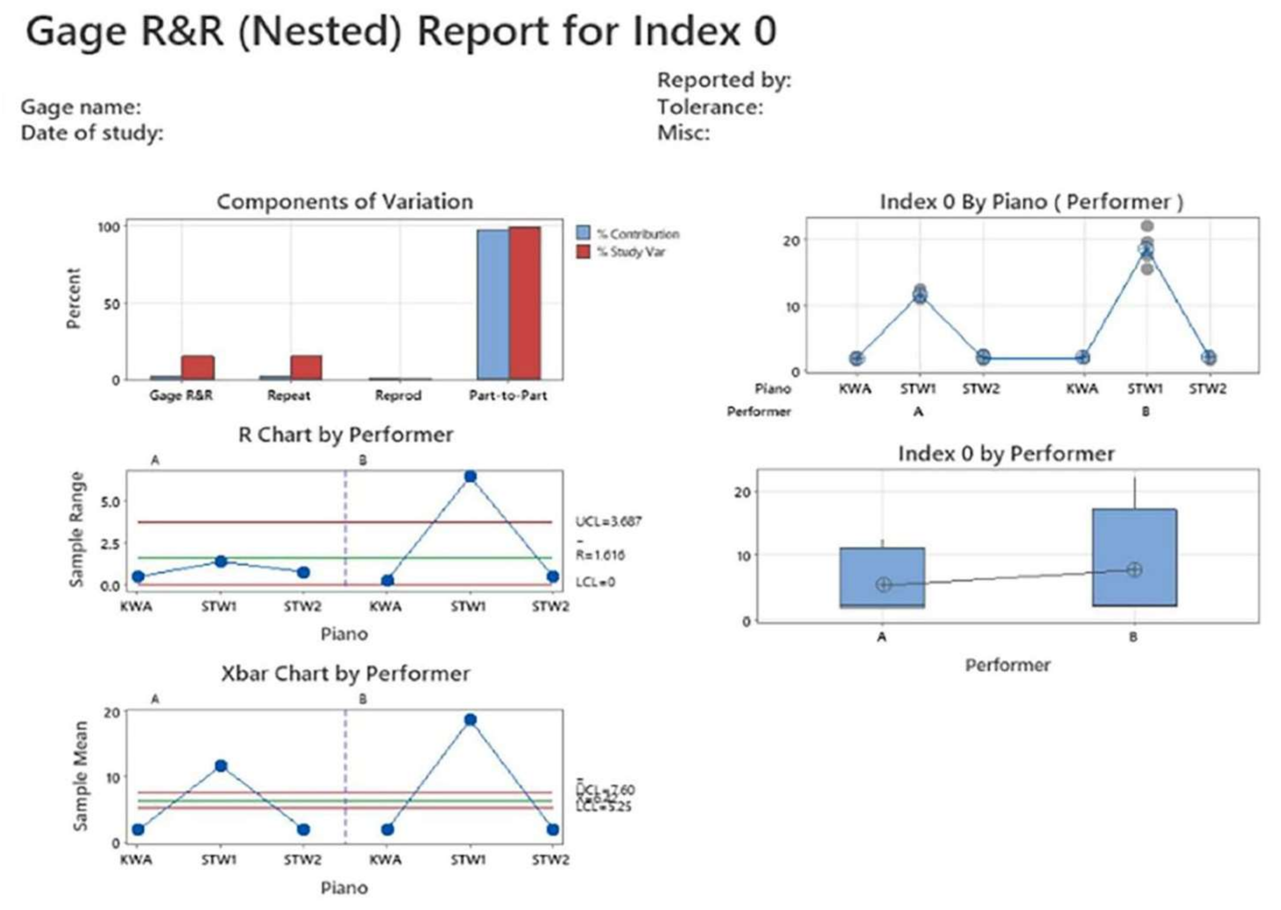

Supplementary Figure 1. Gage R&R Analysis Report for Index 0 Energy Integral. The %SV result was 15 (see Components of Variation Chart), indicating marginally acceptable precision. R Chart has one point above upper control limit (UCL), indicating repeatability is not good at that point. Xbar chart showed points spreading well beyond the UCL and lower control limit (LCL), indicating Index 0 can well differentiate timbre from different pianos. Index 0 by Performer chart and Index 0 by Piano (Performer) chart showed some differences between performer A and B results, indicating reproducibility is not good.

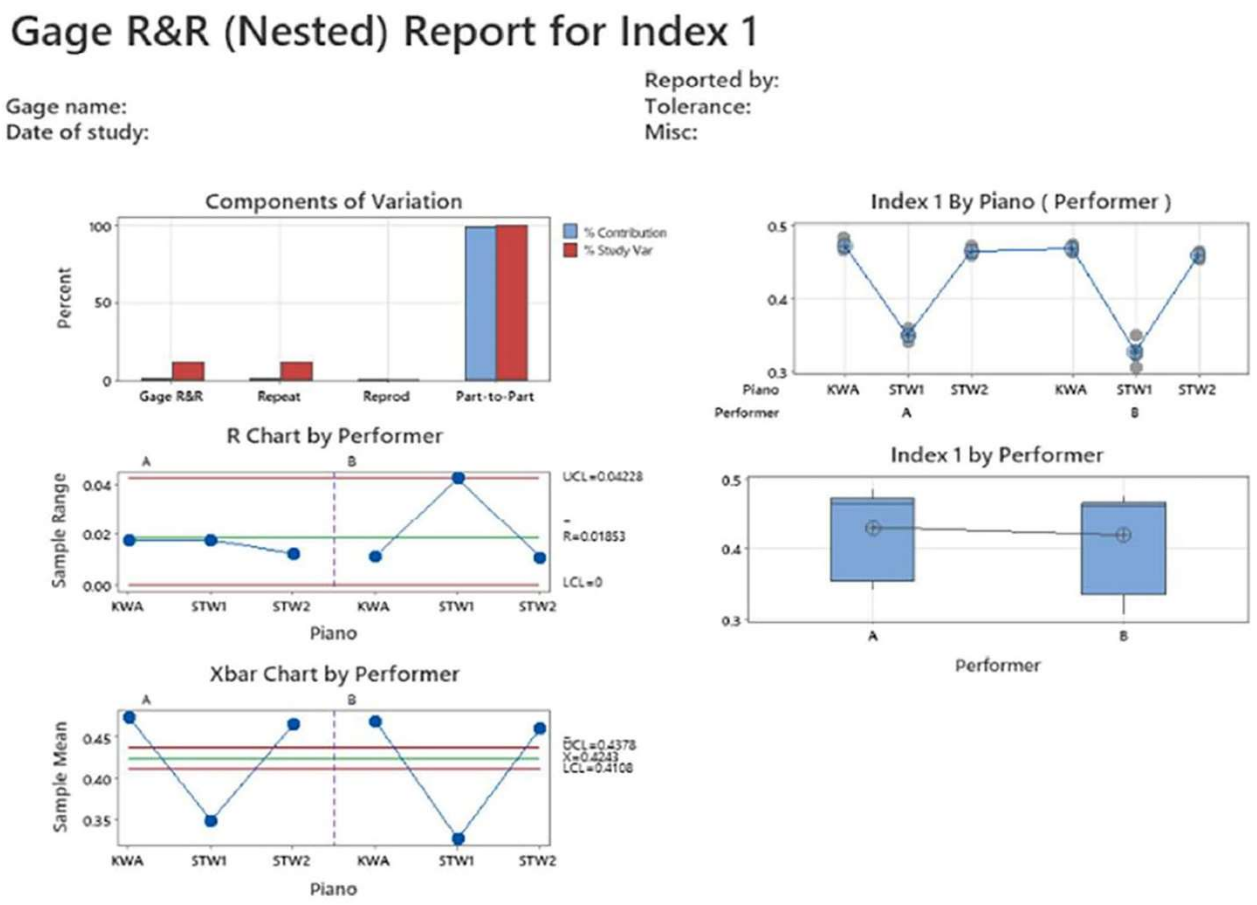

Supplementary Figure 2. Gage R&R Analysis Report for Index 1 Harmonic Mean of Relative Volume. The %SV result was 12.06 (see Components of Variation Chart), indicating marginally acceptable precision. R Chart has one point slightly above UCL, indicating repeatability is not good at that point. Xbar chart showed points spreading well beyond the UCL and LCL, indicating Index 1 can well differentiate timbre from different pianos. Index 1 by Performer chart and Index 1 by Piano (Performer) chart showed some differences between performer A and B results, indicating reproducibility is not good.

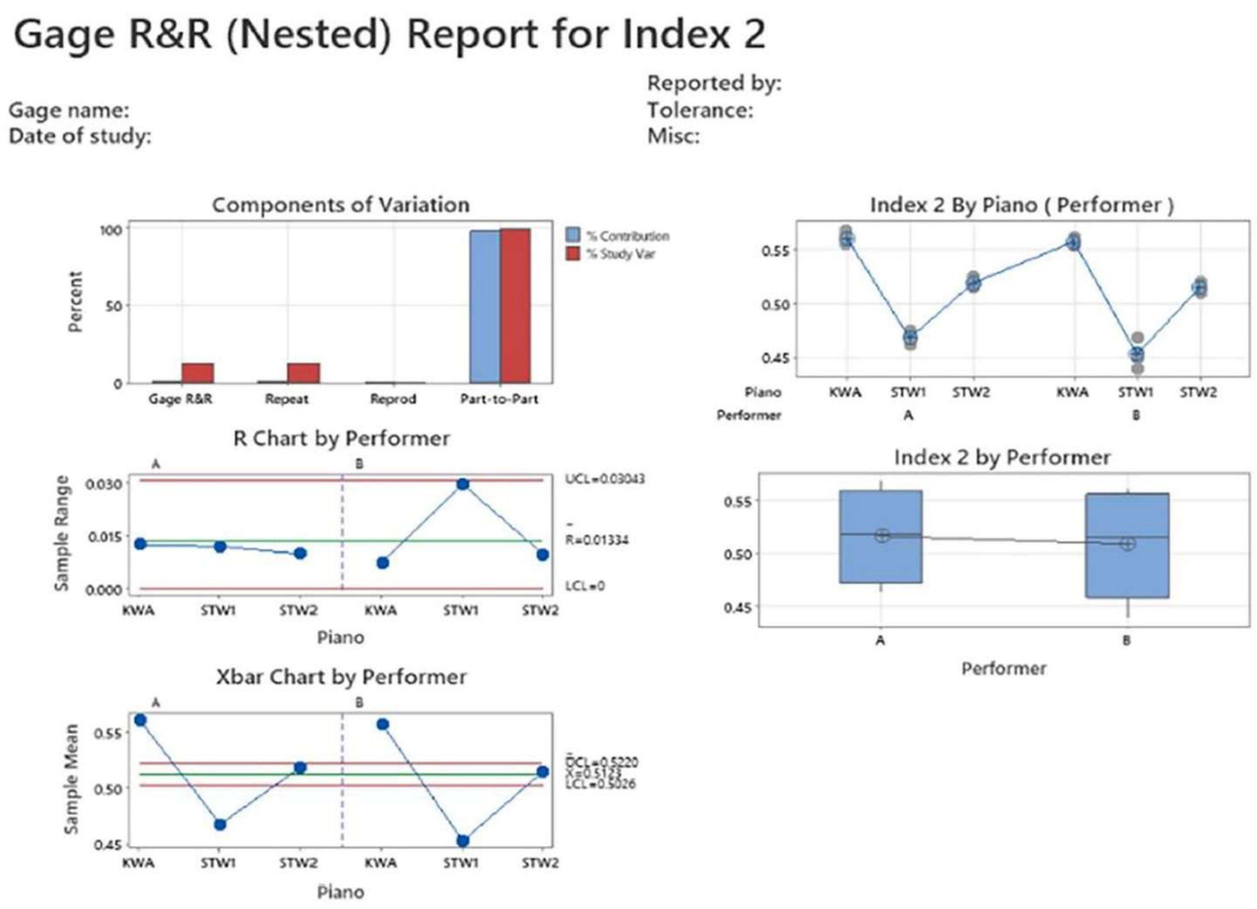

Supplementary Figure 3. Gage R&R Analysis Report for Index 2 Arithmetic Mean of Relative Volume. The %SV result was 12.84 (see Components of Variation Chart), indicating marginally acceptable precision. R Chart has one point lying close to UCL, but all points fell within UCL and LCL, indicating repeatability is okay. Xbar chart showed points spreading well beyond the UCL and LCL, indicating Index 2 can well differentiate timbre from different pianos. Index 2 by Performer chart and Index 2 by Piano (Performer) chart showed some differences between performer A and B results, indicating reproducibility is not good.

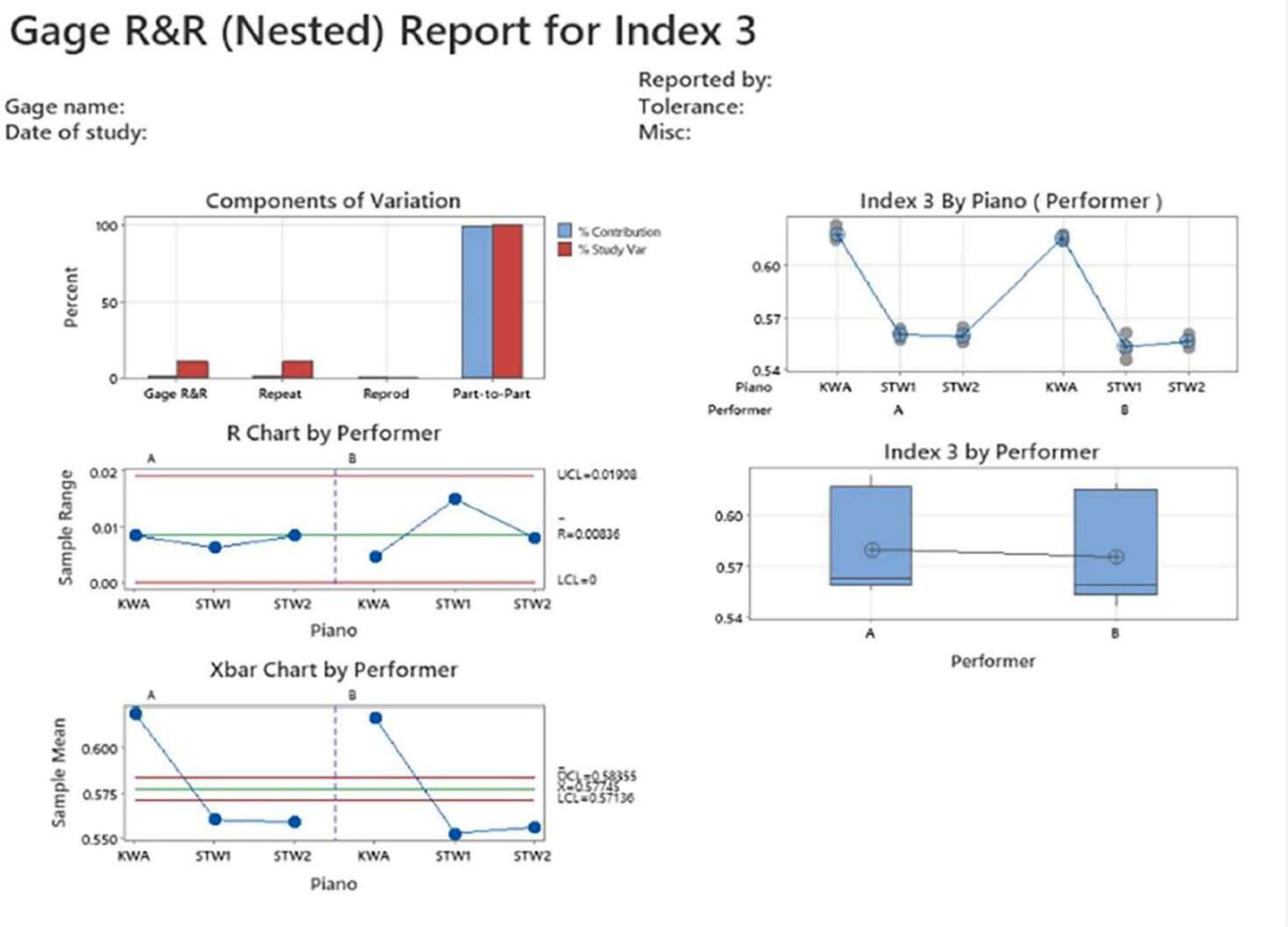

Supplementary Figure 4. Gage R&R Analysis Report for Index 3 Relative Volume RMS. The %SV result was 10.76 (see Components of Variation Chart), indicating marginally acceptable precision. R Chart has all points falling within UCL and LCL, indicating repeatability is good. Xbar chart showed points spreading well beyond the UCL and LCL, indicating Index 3 can well differentiate timbre from different pianos. Index 3 by Performer chart and Index 3 by Piano (Performer) chart showed slight differences between performer A and B results, indicating reproducibility is not very good.

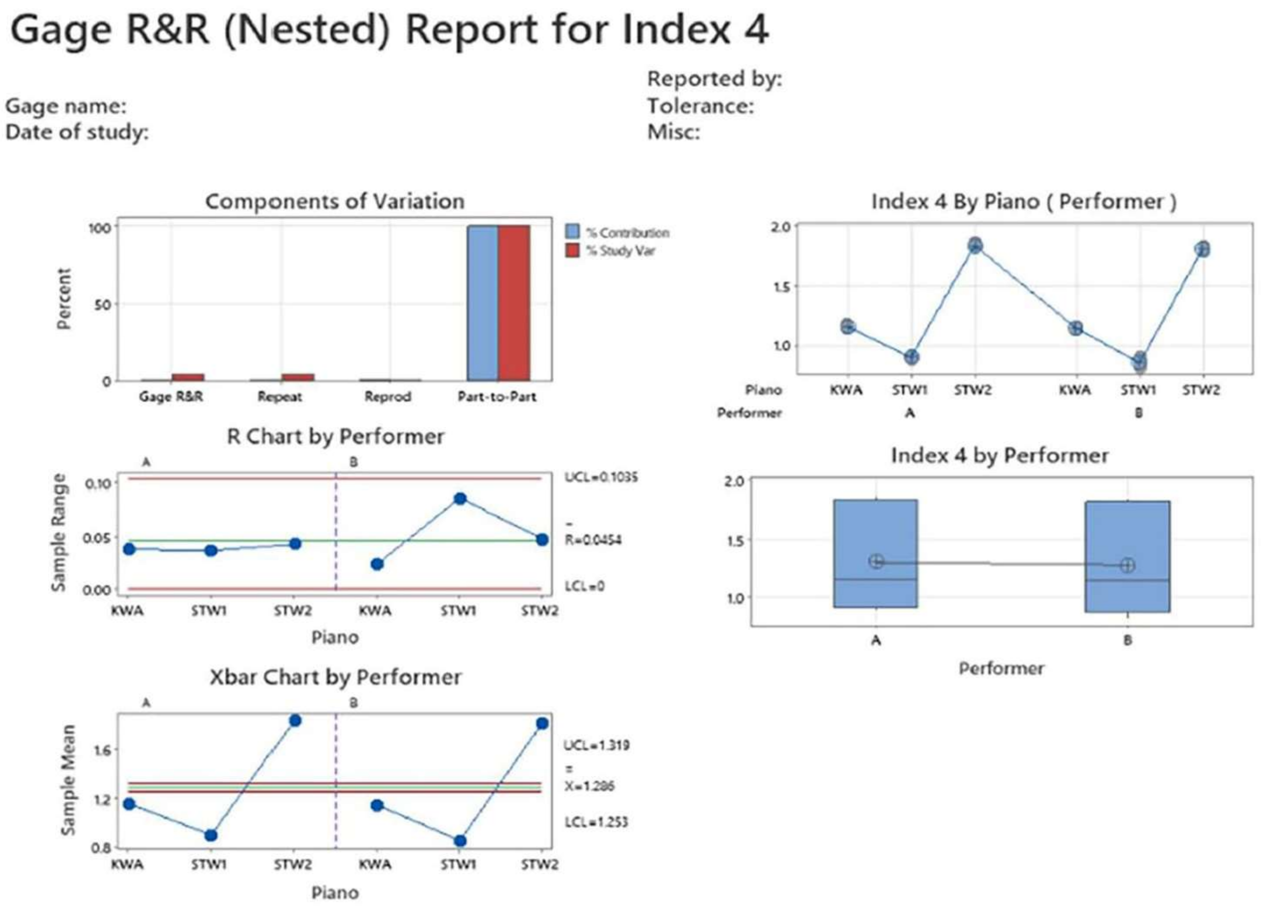

Supplementary Figure 5. Gage R&R Analysis Report for Index 4 Frequency Weighted Arithmetic Mean of Relative Volume. The %SV result was 4.29 (see Components of Variation Chart), indicating acceptable precision. R Chart has all points falling within UCL and LCL, indicating repeatability is good. Xbar chart showed points spreading well beyond the UCL and LCL, indicating Index 4 can well differentiate timbre from different pianos. Index 4 by Performer chart and Index 4 by Piano (Performer) chart showed almost identical results between performer A and B, indicating reproducibility is good.

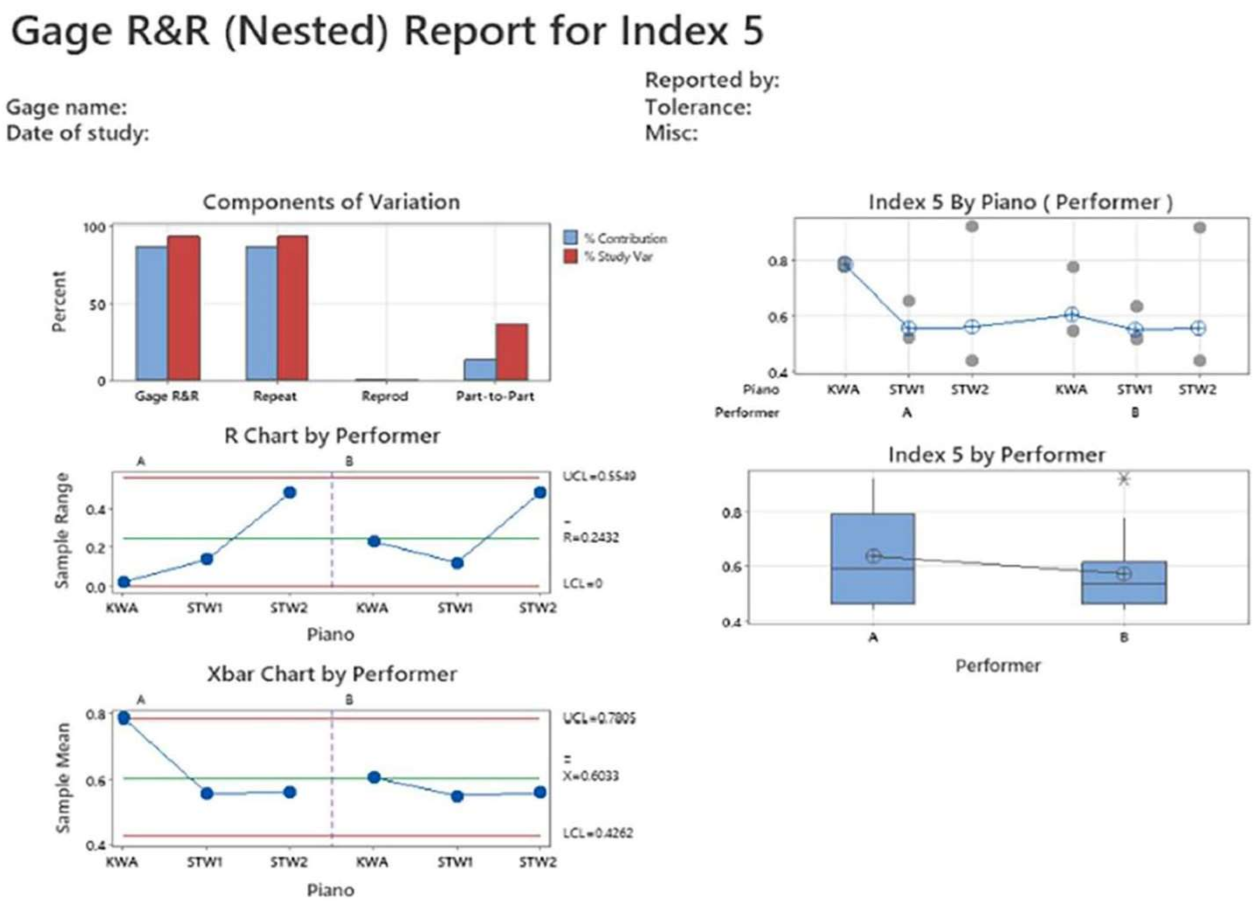

Supplementary Figure 6. Gage R&R Analysis Report for Index 5 Frequency Weighted Relative Volume RMS. The %SV result was 93.12 (see Components of Variation Chart), indicating unacceptable precision. R Chart has all points fell within UCL and LCL, indicating no result for a piano had particularly worse repeatability than others, but repeatability is not good across all pianos, as shown by the scattered measurement result points in the Index 5 by Piano (Performer) chart. Xbar chart showed points falling within the UCL and LCL, indicating Index 5 cannot well differentiate timbre from different pianos. Index 5 by Performer chart and Index 5 by Piano (Performer) chart showed differences between performer A and B results, indicating reproducibility is not good.

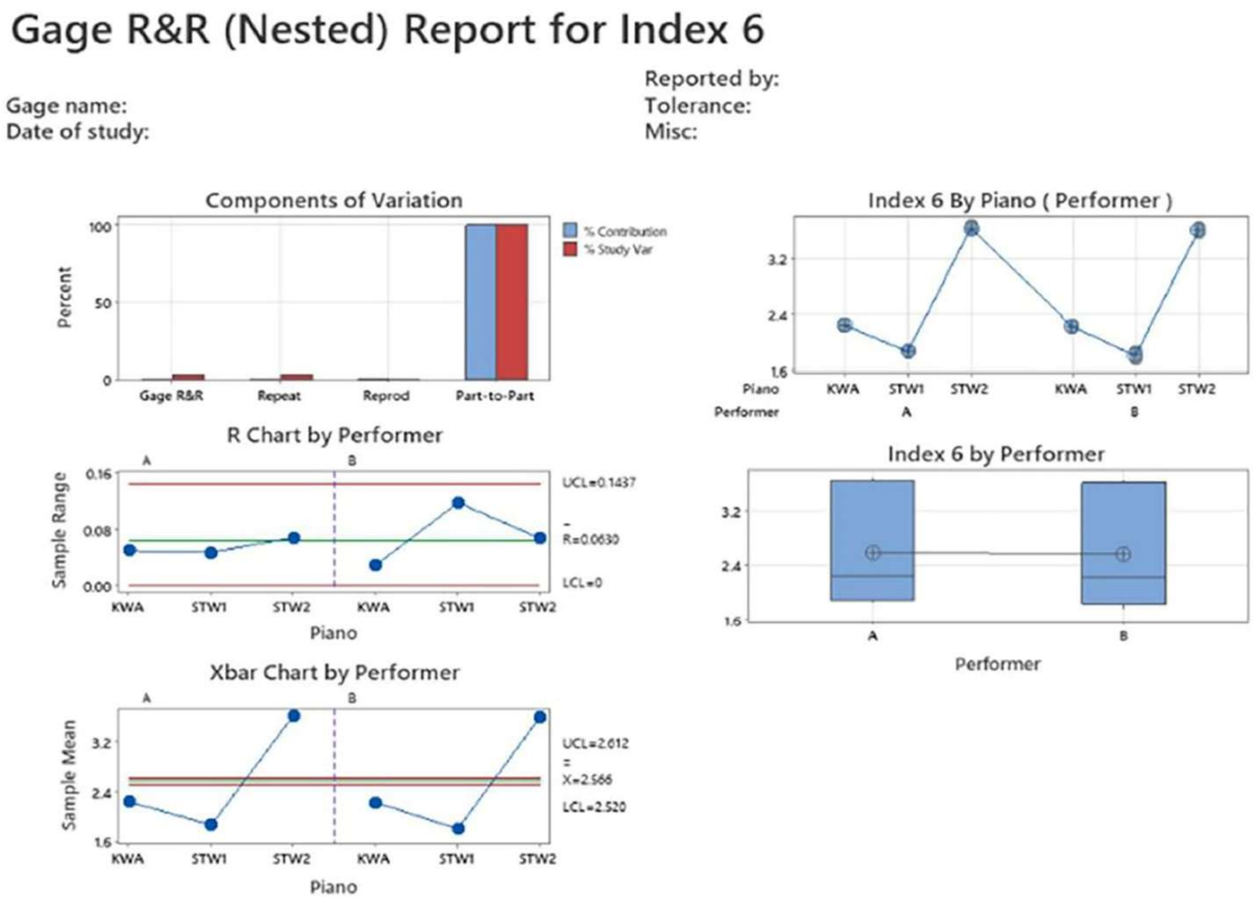

Supplementary Figure 7. Gage R&R Analysis Report for Index 6 Sum of Relative Volume. The %SV result was 3.11 (see Components of Variation Chart), indicating acceptable precision. R Chart has all points falling within UCL and LCL, indicating repeatability is good. Xbar chart showed points spreading well beyond the UCL and LCL, indicating Index 6 can well differentiate timbre from different pianos. Index 6 by Performer chart and Index 6 by Piano (Performer) chart showed almost identical results between performer A and B, indicating reproducibility is good.

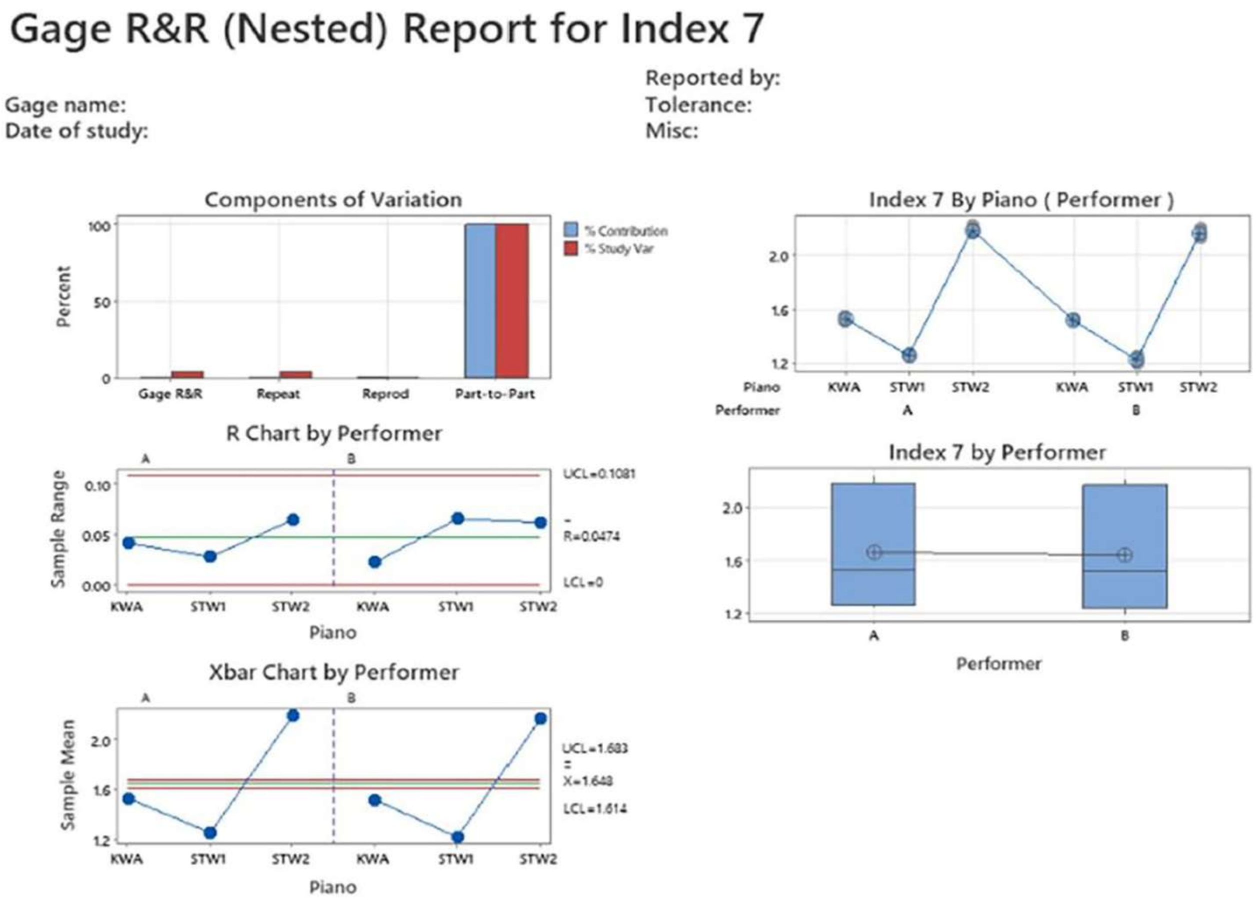

Supplementary Figure 8. Gage R&R Analysis Report for Index 7 Square Sum of Relative Volume. The %SV result was 4.4 (see Components of Variation Chart), indicating acceptable precision. R Chart has all points falling within UCL and LCL, indicating repeatability is good. Xbar chart showed points spreading well beyond the UCL and LCL, indicating Index 7 can well differentiate timbre from different pianos. Index 7 by Performer chart and Index 7 by Piano (Performer) chart showed almost identical results between performer A and B, indicating reproducibility is good.

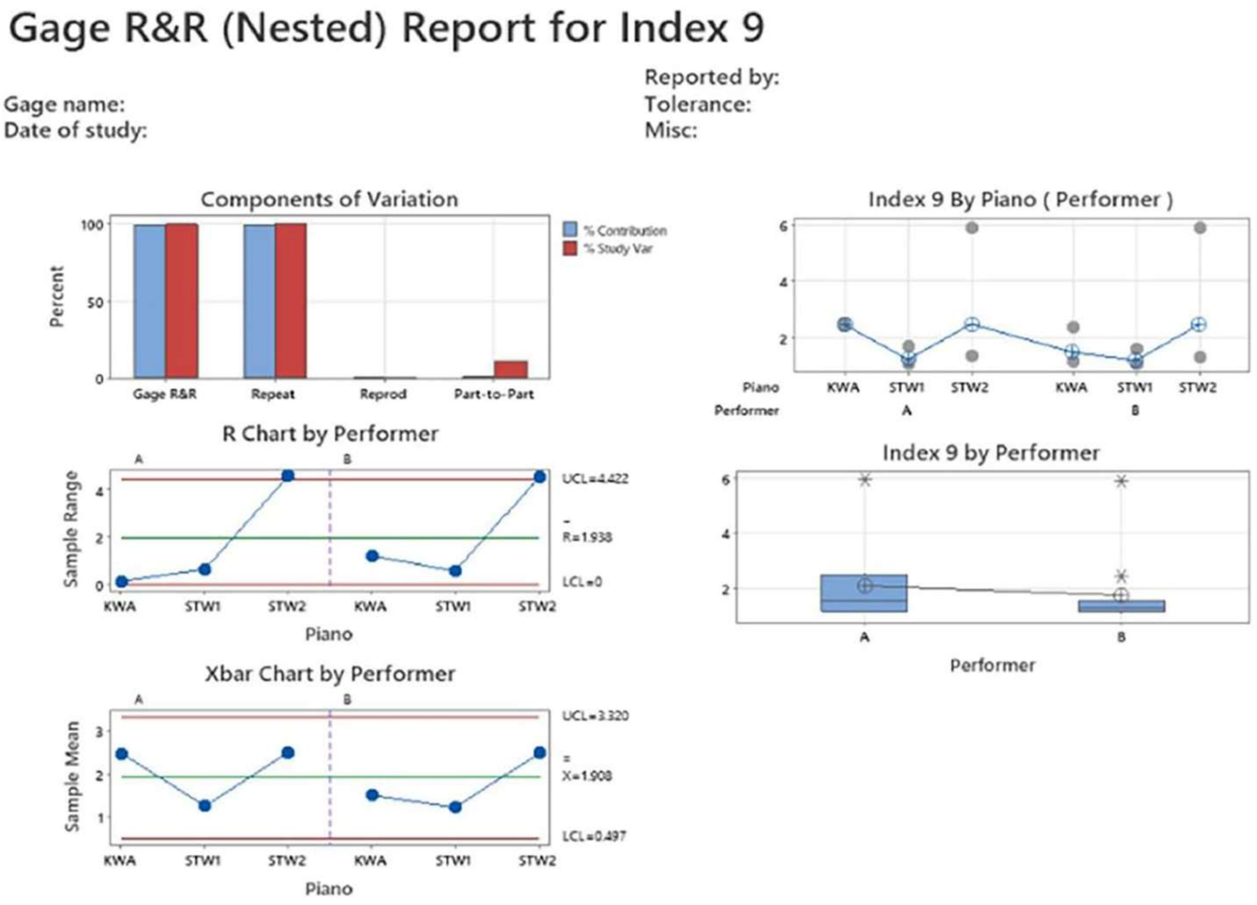

Supplementary Figure 9. Gage R&R Analysis Report for Index 9 Frequency Weighted Square Sum of Relative Volume. The %SV result was 99.43 (see Components of Variation Chart), indicating unacceptable precision. R Chart has has two points lying beyond UCL, indicating repeatability is not good. Xbar chart showed points falling within the UCL and LCL, indicating Index 9 cannot well differentiate timbre from different pianos. Index 9 by Performer chart and Index 9 by Piano (Performer) chart showed differences between performer A and B results, indicating reproducibility is not good.
